# Identifying Neuropeptide and G Protein-Coupled Receptors of Juvenile Oriental River Prawn (*Macrobrachium nipponense*) in Response to Salinity Acclimation

**DOI:** 10.3389/fendo.2020.00623

**Published:** 2020-09-08

**Authors:** Shengming Sun, Mengru Zhu, Fangyan Pan, Jianbin Feng, Jiale Li

**Affiliations:** ^1^Key Laboratory of Exploration and Utilization of Aquatic Genetic Resources, Shanghai Ocean University, Ministry of Education, Shanghai, China; ^2^International Research Center for Marine Biosciences at Shanghai Ocean University, Ministry of Science and Technology, Shanghai, China; ^3^Wuxi Fishery College, Nanjing Agricultural University, Wuxi, China

**Keywords:** *Macrobrachium nipponense*, neuropeptides, salinity, GPCRs, eyestalk

## Abstract

Neuropeptides and their G protein-coupled receptors (GPCRs) from the central nervous system regulate the physiological responses of crustaceans. However, in crustaceans, our knowledge regarding GPCR expression patterns and phylogeny is limited. Thus, the present study aimed to analyze the eyestalk transcriptome of the oriental river prawn *Macrobrachium nipponense* in response to salinity acclimation. We obtained 162,250 unigenes after *de novo* assembly, and 1,392 and 1,409 differentially expressed genes were identified in the eyestalk of prawns in response to low and high salinity, respectively. We used combinatorial bioinformatic analyses to identify *M. nipponense* genes encoding GPCRs and neuropeptides. The mRNA levels of seven neuropeptides and one GPCR were validated in prawns in response to salinity acclimation using quantitative real-time reverse transcription polymerase chain reaction. A total of 148 GPCR-encoding transcripts belonging to three classes were identified, including 77 encoding GPCR-A proteins, 52 encoding GPCR-B proteins, and 19 encoding other GPCRs. The results increase our understanding of molecular basis of neural signaling in *M. nipponense*, which will promote further research into salinity acclimation of this crustacean.

## Introduction

Crustacean culture provides high-quality food as well as huge economic benefits to farmers and the economy. Among them, the *Macrobrachium nipponense* is an economically important economic species in aquaculture, with a production of in excess of 250,000 tons and an output reaching 2 billion RMB per year in China (1). In the aquaculture industry, culturing seawater species for desalination and using freshwater crustacean species for saltwater acclimation are new trends (2). In the past two decades, large numbers of the genus *Macrobrachium* have invaded freshwater habitats from the ancestral marine environment, and have exhibited high adaptability to slightly brackish and freshwater habitats (3–5). However, to date, few studies have investigated the mechanisms that regulate salinity adaptation in *M. nipponense*.

Salinity is an important environmental factor in estuarine and coastal systems, which affects the physiology of crustaceans and determines species distributions (6). There is a growing interest in improving prawn performance in aquaculture at low salinity. Previous studies have confirmed that a number of key neuropeptides participate in salinity stress responses of crustacean (7, 8). Neuropeptides mostly bind to G protein-coupled receptors (GPCRs) on the cell surface (9). GPCRs, as seven-pass integral membrane proteins, play key roles as transducers of extracellular signals across the lipid bilayer (10, 11), and act as salinity sensors in aquatic animal (12). Thus, the identification of neuropeptides and GPCRs represents an essential step to unraveling the roles of these molecules in response to salinity acclimation.

Rapid developments in RNA sequencing make it possible to use bioinformatics approaches to identify neuropeptides and their cognate GPCRs. Although neuropeptide sequences have been identified using *in silico* transcriptome analysis in many crustaceans (13–16), no information to date was provided to identify neuropeptides and GPCRs from eyestalk tissues of female *M. nipponense* during salinity acclimation, especially, knowledge of the GPCRs is limited in crustacean. In the present study, we aimed to perform gene expression profile analysis (control vs. low salinity group and control vs. high salinity group) to identify neuropeptides and GPCRs from eyestalk tissues of prawns responded to salinity stress. We also aimed to validate target transcripts encoding for neuropeptides and their cognate that might have important functions in *M. nipponense* salinity adaptation. The results will provide insights into salinity-mediated regulation of neuropeptide/GPCR signaling pathways in *M. nipponense*.

## Materials and Methods

### Experimental Animals and Salinity Treatment

Juvenile *M. nipponense* specimens were obtained from a farm in Shanghai (Qingpu) and acclimated to laboratory conditions for 14 days in fresh water (temperature 22 ± 1°C, pH 7.7 ± 0.6, dissolved oxygen content 6.5 ± 0.5 mg/L). Thereafter, 360 healthy prawns (1.82 ± 0.46 g wet weight) were randomly and equally divided into 12 tanks (30 per tank), and the tanks were randomly assigned to three groups (three tanks per group). The salinity was gradually adjusted on the same day to reach the target salinity for each group: S0 = 0.4 (control group), S6 = 8 ± 0.2 (low salinity), S12 = 16 ± 0.2 (high salinity). Salinity and water quality were maintained as previously described (2), and the prawns provided with commercial feed (Zhejiang Tongwei Feed Group CO., Ltd) twice daily for 1 week at a ratio of 6–8% of their body weight.

### Identification of Neuropeptides and Their Putative Cognate GPCRs

Total RNA extraction from nine prawns in each group, RNA-Seq library preparation and sequencing were carried out based on Illumina HiSeq™ 2500 paired-end sequencing technology, as previously described (17). Trinity was used to assemble a transcriptome data from eyestalk tissues and generated the unigenes. All unigenes were annotated based on the NCBI databases with a cut-off E value of 1.0 × 10^−5^, Further, the BLAST2GO program was used for GO analysis (http://www.geneontology.org/), and Clusters of Orthologous Groups (COG) classification and signal pathway annotation of unigenes was performed by conducting BLASTx searches. EdgeR uses a negative binomial distribution method with pairwise test using Fisher for identified differentially expressed genes (DGE) between control and salinity treatment group. Subsequently, GO and Kyoto Encyclopedia of Genes and Genomes (KEGG) pathway classification of DEGs was carried out as previously described (17). The transcriptomic data (NCBI Sequence Read Archive: SRP251206) derived from eyestalk tissue were used to identify neuropeptides and receptors. To search for *M. nipponense* neuropeptides, the annotated sequences and the open reading frame (ORF) file were searched for keywords related to known neuropeptides and for conserved amino acid sequences, respectively (18, 19). Finally, the identified sequences were combined with a list of previously obtaining and characterized neuropeptides (Supplementary Material 1).

The Pfam-v27 module in CLC Genomics Workbench v9.5 (Qiagen, Hilden, Germany) was used to predict the structural domains in the GPCRs (intra/extracellular loops and seven transmembrane domains (7-TM). Bioinformatic analysis was also carried out on previously reported neuropeptide GPCRs from decapods (20, 21). Local BLAST was used to compare the GPCR sequences, followed by clustering analysis using BioLayout Express 3D (22) at an e-value cutoff of 1e-20. All GPCR sequences (those from our data and previously characterized receptors) were then combined into one list (Supplementary Material 2). Then, the GPCRs were multiply aligned using the CLUSTALW algorithm, imported into MEGA 7.0, and subjected to phylogenetic analysis (23, 24).

### Quantitative Real-Time Reverse Transcription PCR

The identification and enrichment analysis of differentially expressed genes (DEGs) were performed according to our previously published methods (17). The cDNAs from salinity treatments of *M. nipponense* were synthesized from total DNA-free RNA (1 μg) using a Prime Script RT reagent kit (TaKaRa, Japan) following the manufacturer's instruction. The Bio-Rad iCycler iQ5 Real Time System (Biorad Inc., Berkeley, CA, USA) was used for qRT-PCR validation of DEGs expression, with the *Actb* gene as the internal control (25). The amplification efficiency and threshold were automatically generated by standard curves. The primer sequences are shown in Supplementary Material 3. The 2^−ΔΔ*CT*^ comparative CT method (26) was used to calculate the relative transcript abundance.

## Results

### Overview of the Transcriptomes

**Figure 1:**
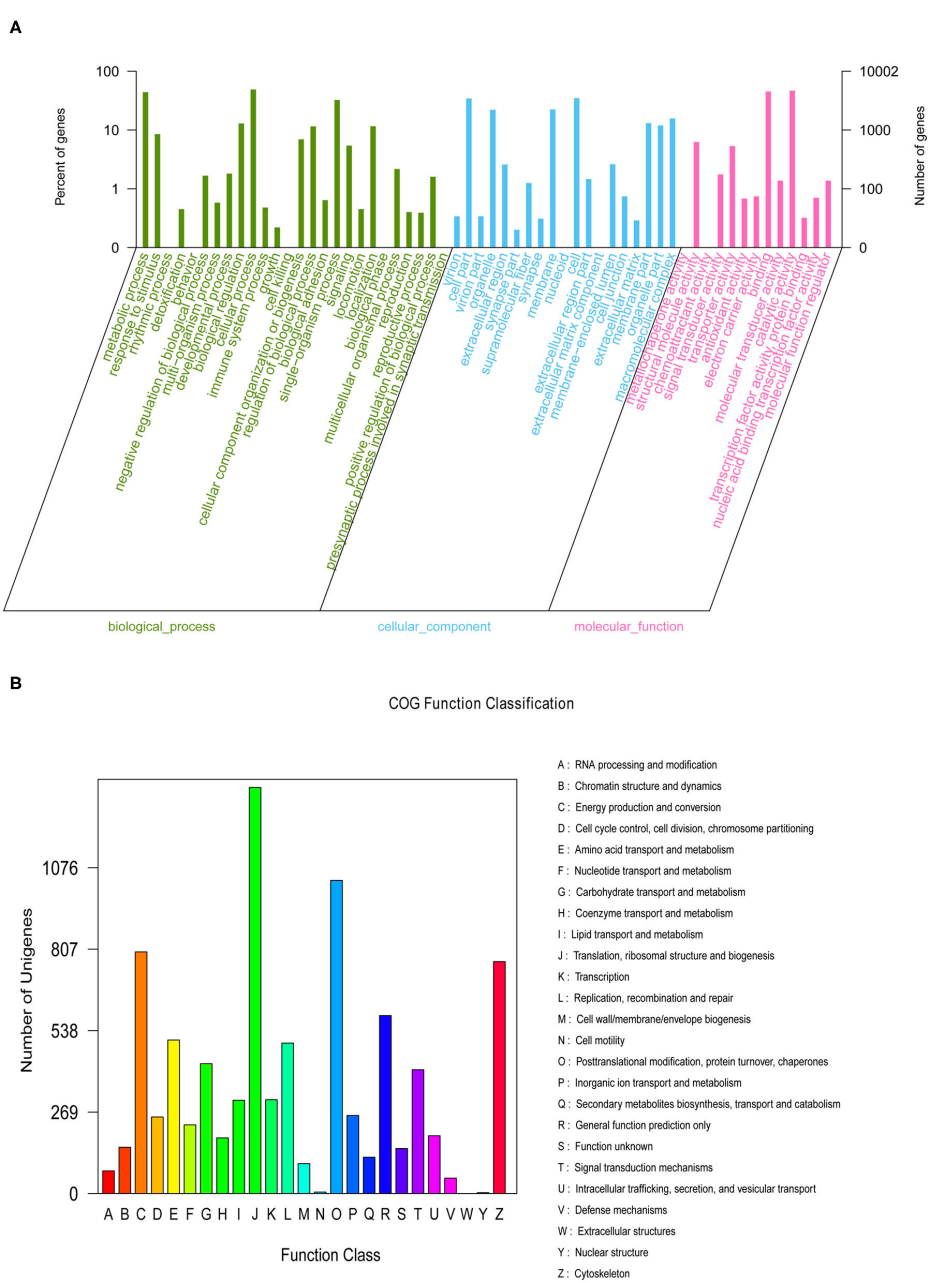
The Gene Ontology **(A)** categorization and Clusters of Orthologous Groups **(B)** functional classification of assembled unigenes.

We generated nine eyestalk transcriptomes in prawns under the three experimental conditions in response to salinity acclimation, including freshwater, low salinity, and high salinity. Analysis using the BUSCO pipeline indicated that >92% of the arthropoda orthologs were present in the assembled transcriptome [Complete BUSCOs (C): 92.6%]. After removing adaptor sequences, ambiguous “N” nucleotides and low quality sequences, a total of 366,728,422 clean reads representing 54,659,786,418 clean nucleotides (nt) were shown in Supplementary Material 4. A total of 162,250 unigenes were obtained for the eyestalk transcriptome. In the GO analysis, 10,002 unigenes were enriched into 58 functional subgroups. Based on COG analysis, 8,755 of the unigenes were allocated to 25 COGs (Figures 1A,B).

### DEGs Identification and Functional Analysis

**Figure 2:**
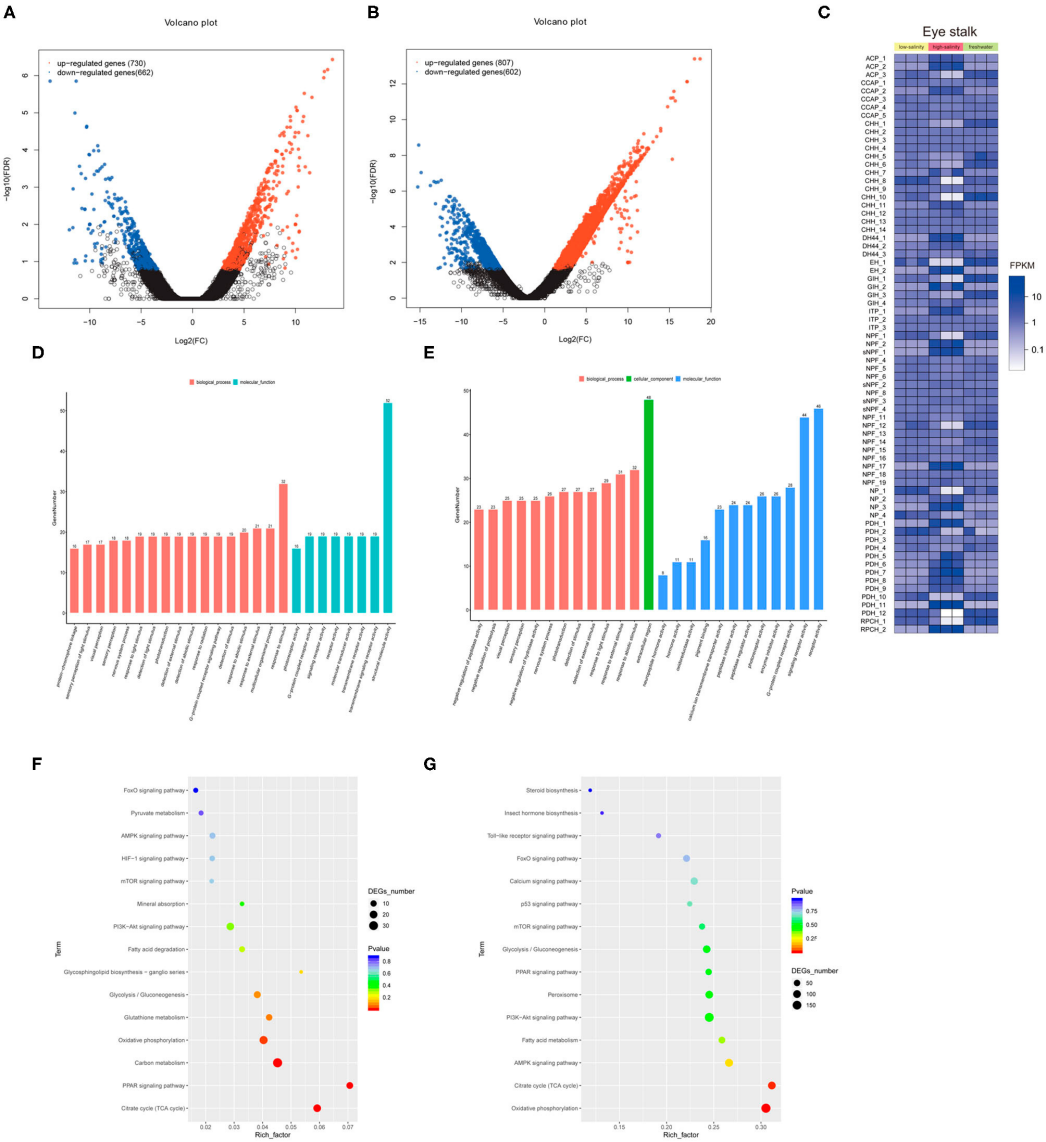
Comparative transcriptome analysis explores neuropeptides from eyestalk in juvenile *M. nipponense* in response to salinity acclimation. Analysis of differentially expressed genes identified in the control vs. the low salinity group **(A)**, and the control vs. the high salinity group **(B)**. Notes: Upregulated and downregulated differentially expressed genes are shown in red and blue, respectively. The X- and Y-axes show the log_2_-fold change and the log_10_ *P*-value of the normalized expression level (Fragments Per Kilobase of transcript per Million mapped reads) of a gene between the two compared groups, respectively. Overview of putative neuropeptides identified in *M. nipponense*. **(C)**. Overview of identified differential expressed neuropeptides in *M. nipponense*. ACP, Adipokinetic hormone/Corazonin-related peptide; CCAP, Crustacean cardioactive peptide; CHH, Crustacean hyperglycemic hormone; EH, Eclosion hormone; GIH, Gonad inhibiting hormone; ITP, Ion transport protein; NP, Neuroparsin; NPF, Neuropeptide F; PDH, Pigment dispersing hormone; sNPF, short neuropeptide F. Top 25 GO classification analyses of DEGs in the control vs. the low salinity group **(D)**, the control vs. the high salinity group **(E)**. Top 15 significant KEGG pathway classifications of DEGs are shown for the control vs. the low salinity group **(F)**, the control vs. the high salinity group **(G)**.

We identified 1,392 and 1,409 genes that were differentially expressed under low salinity and high salinity, respectively (Figures 2A,B). The heat map of identified putative neuropeptide precursors and their RNA-seq FPKM expression levels, were compared between freshwater culture and salinity acclimation, such as CCAP, crustacean hyperglycemic hormone (CHH) and ion transport peptide (ITP), and so on (Figure 2C). The biological functions of the DEGs were determined using GO functional annotation (Figures 2D,E), which were significantly over-represented (*p* < 0.05, FDR < 0.01) as shown “G-protein coupled receptor signaling pathway” (GO:0007186), “response to external stimulus” (GO:0009605). In addition, KEGG pathway enrichment analysis identified the 15 most significant pathways (Q < 0.05) associated with salinity acclimation (Figures 2F,G), both including represented metabolism pathway “Glycolysis/Gluconeogenesis,” “Citrate cycle,” and “Fatty acid metabolism.”

### Bioinformatic Identification of Putative GPCRs

**Figure 3:**
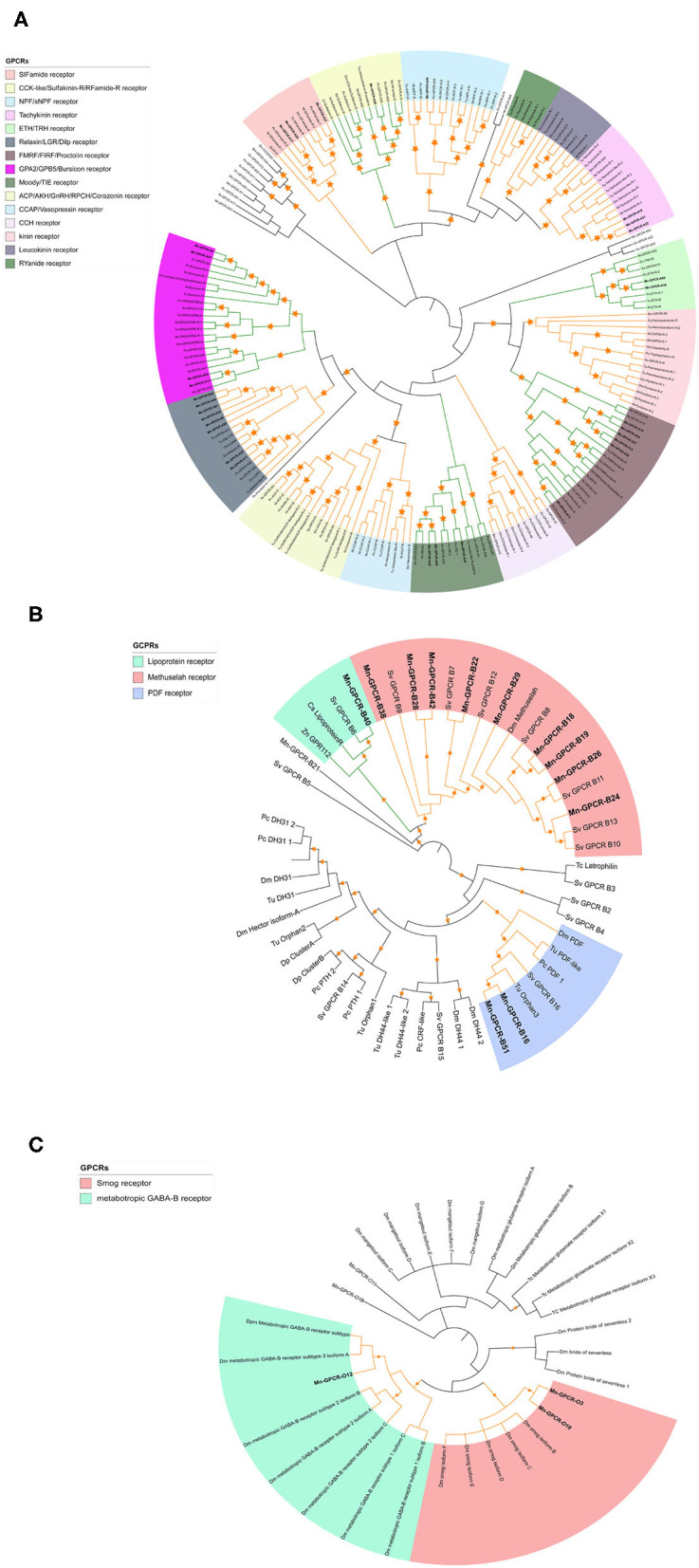
Phylogenetic tree of Rhodopsin class GPCRs [GPCRs (GPCR-As, **(A)**], Secretin class GPCRs [(GPCR-Bs, **(B)**] and other GPCRs **(C)** of *M. nipponense* and other invertebrate species. Orange stars represent clades with a bootstrap value larger than 70. Green line: Clade annotated with high confidence. Yellow line: Clade annotated with low confidence. Red line: Unannotated clade. Bm, *Bombyx mori*; Dp, *Daphnia pulex*; Dm, *Drosophila melanogaster*; Nn, *Nephrops norvegicus*; Nl, *Nilaparvata lugens*; Pc, *Procambarus clarkii*; Sv, *Sagmariasus verreauxi*; Tu, *Tetranychus urticae*; Zn, *Zootermopsis nevadensis*; Tc, *Tribolium castaneum*.

Clustering and phylogenetic analyses identified 223 putative GPCR genes based on the *de novo* nine transcriptome datasets. Phylogenetic analysis showed that 34 of the GPCRs could be classified as GPCR-A proteins (Figure 3A), which included receptors for red pigment concentrating hormone (RPCH), adipokinetic hormone-related neuropeptide/corazonin-related peptide (ACP), and CCAP. Forty-four of the putative GPCRs were classified as GPCR-B proteins (Figure 3B). Three putative GPCR families within the GPCR-B classification were identified using comparative phylogenetics with high-confidence, including the lipoprotein receptor, methuselah receptor, and pigment dispersing hormone (PDH) receptor. The third group comprised the remaining uncharacterized GPCR families (Figure 3C), for example the metabotropic GABA-B receptor and smog receptor.

### Verification Neuropeptide Expression

**Figure 4:**
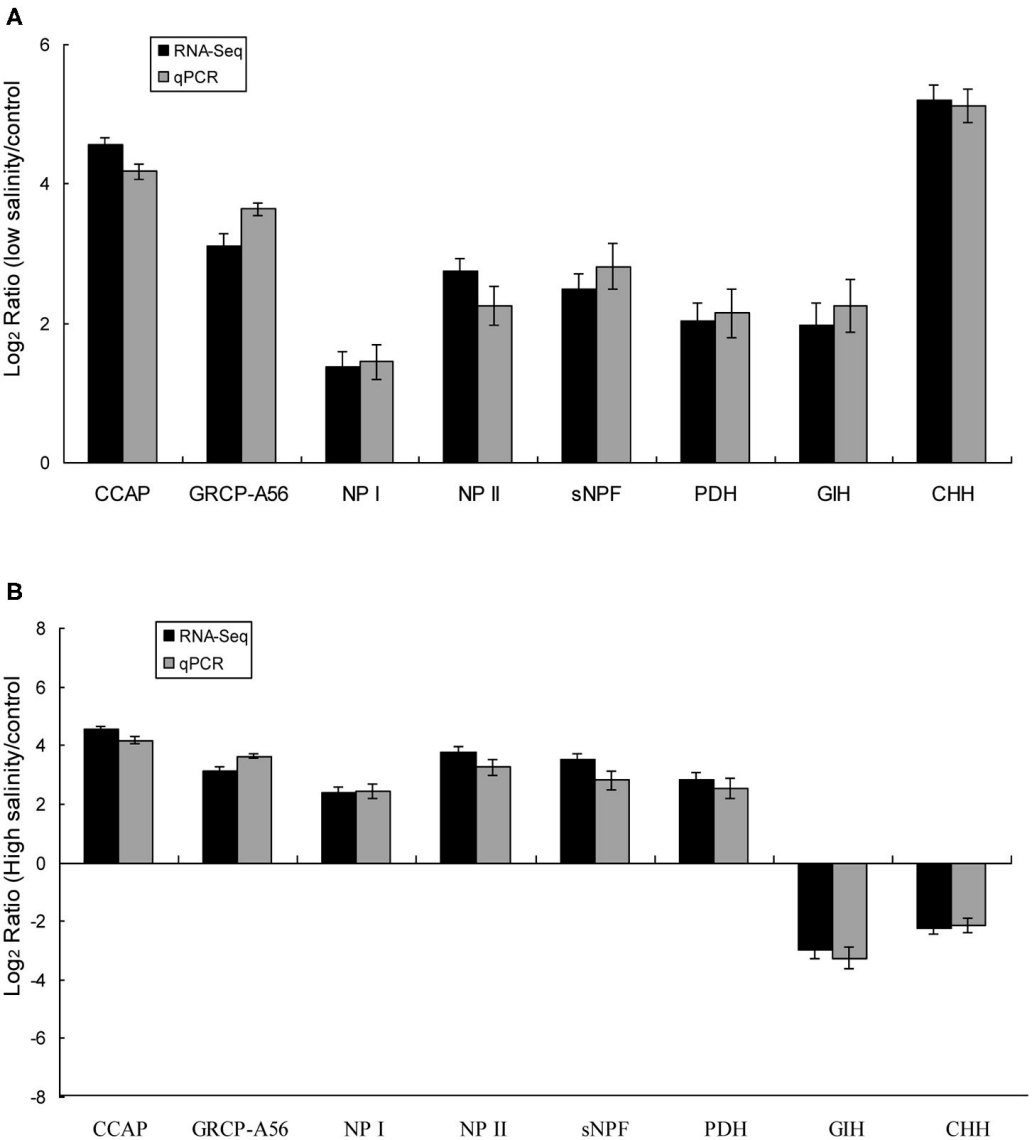
Validation of the identified DEGs using qRT-PCR in the control vs. the low salinity group **(A)**, the control vs. the high salinity group **(B)**. The X-axis represents genes in the eyestalk tissue validated in this study; the Y-axis shows the Log_2_ Ratio of expression in *M. nipponense* in response to salinity acclimatization. No significant differences were found between the qRT-PCR and Illumina data (*R* = 0.95).

Eight predicted significant DEGs encoding neuropeptides were identified, including those encoding isoforms of CCAP, CHH, short neuropeptide F (sNPF), PDH, gonad-inhibiting hormone (GIH), and neuroparsins (NP), as well as a CCAP receptor (GRCP-A56). The expression trends of the eight DEGs identified in eyestalk of prawns in response to salinity acclimation from the RNA-seq data were verified using RT-PCR (Figures 4A,B). The expression levels of the eight DEGs were significantly higher in the low salinity group compared with that in the control group. By contrast, two DEGs (encoding GIH and CHH) showed the opposite trend in the high salinity group compared with that in the control group. Additionally, DEGs encoding CCAP, GRCP-A56, sNPF, NP I, NP, II, and PDH were significantly upregulated in the high salinity group.

## Discussion

The assembled transcriptome contained sequences representing 52 different neuropeptide precursors, most of which are present in other crustacean species. Importantly, our study was the first to indicate that certain neuropeptides in prawns play an important role in response to salinity acclimation. Interestingly, some neuropeptide transcripts that were detected previously in other decapod crustacean species were not identified in this *M. nipponense* transcriptome, such as crustacean female sex hormone (CFSH) (27, 28). Notably, our previous *M. nipponense de novo* transcriptome assembly did include these neuropeptides, which partially disagrees with the results of the present study. A reasonable explanation is that differences in the identified neuropeptides were closely related to crustacean habitats (freshwater vs. estuary) and developmental stage (adult vs. larval). Data analysis predicted 148 different GPCRs, which is similar to the number predicted in *Chilo suppressalis* (29). A lack of close homologs of known function from related species made confident annotation of these GPCRs difficult. In addition, certain neuropeptide GPCRs identified previously in other arthropods (e.g., Crz, sulfakinin, and pyrokinin receptors) were not observed on the present phylogenetic analysis.

KEGG analysis identified energy metabolism pathways that were significantly affected by salinity, such as glycometabolism, which were similar to previous study in *Litopenaeus vannamei* (30), our further study will focus on the aspects of energy metabolism of prawns under salinity acclimation. Interestingly, GO functional annotation of the DEGs was associated with “G-protein coupled receptor signaling pathway” of prawns responded to salinity acclimation. Thus, we identified differentially expressed neuropeptides and GPCRs genes, which are plausibly related to salinity acclimation. The neuropeptides and their putative cognate receptors were analyzed using qRT-PCR. For example, CCAP is a C-terminal amidated non-apeptide hormone found in many crustacean species, such as blue crab (*Callinectes sapidus*) (31). In addition to its role in heartbeat regulation, direct evidence points to a role for CCAP in the regulation of homeostasis in *L. vannamei* (8), which was consistent with our results that CCAP and its receptor mRNA expression was upregulated under high- and low-salinity conditions in *M. nipponense*.

In agreement with the results of the present study, previous studies confirmed that salinity changes in crustaceans upregulated the transcript levels of peptide hormones (32, 33), such as CHH and ITP. The injection of purified CHH increased the Na+ concentration and osmolality in the hemolymph (34). Notably, crustacean CHHs showed high sequence homology to ITP (35). Our results indicated much higher levels of ITP transcripts in the high salinity and low salinity groups than in the control group, suggesting that ITP might function in ionic transport or osmo-regulation, or both, in prawns. GIH has an important function in crustacean ovarian maturation inhibition (36). The results of the present study showed that high salinity downregulated GIH expression. This indicated that salinity and gonadal development might correlate strongly in *M*. *nipponense*. Therefore, further study is required to gain a better understanding of the functions of these neuropeptides and their GPCRs associated with the effects of salinity on the prawn reproduction system.

## Data Availability Statement

All datasets generated for this study are included in the article/Supplementary Material.

## Author Contributions

SS and JL conceived, designed the experiments, and supervised the project. SS, MZ, FP, and JF carried out the experiments and analyzed the data. SS wrote the manuscript.

## Conflict of Interest

The authors declare that the research was conducted in the absence of any commercial or financial relationships that could be construed as a potential conflict of interest.

## Supplementary Material

Supplementary Material 1 Comparative list of neuropeptides detected in previous studies.

Supplementary Material 2 All GPCRs sequences used in this study.

Supplementary Material 3 The specific primers used for qRT-PCR.

Supplementary Material 4 Summary of sequencing and functional annotation of unigenes.
